# Properties and Molecular Determinants of the Natural Flavone Acacetin for Blocking hKv4.3 Channels

**DOI:** 10.1371/journal.pone.0057864

**Published:** 2013-03-20

**Authors:** Hui-Jun Wu, Hai-Ying Sun, Wei Wu, Yan-Hui Zhang, Guo-Wei Qin, Gui-Rong Li

**Affiliations:** 1 Department and Medicine, Li Ka Shing Faculty of Medicine, The University of Hong Kong, Pokfulam, Hong Kong, China; 2 Shanghai Institute of Materia Medica, Chinese Academy of Science, Shanghai, China; 3 Department of Physiology, Li Ka Shing Faculty of Medicine, The University of Hong Kong, Pokfulam, Hong Kong, China; Centro Nacional de Biotecnologia-CSIC, Spain

## Abstract

The natural flavone acacetin has been demonstrated to inhibit transient outward potassium current (I_to_) in human atrial myocytes. However, the molecular determinants of acacetin for blocking I_to_ are unknown. The present study was designed to investigate the properties and molecular determinants of this compound for blocking hKv4.3 channels (coding I_to_) stably expressed in HEK 293 cells using the approaches of whole-cell patch voltage-clamp technique and mutagenesis. It was found that acacetin inhibited hKv4.3 current by binding to both the closed and open channels, and decreased the recovery from inactivation. The blockade of hKv4.3 channels by acacetin was use- and frequency-dependent, and IC_50_s of acacetin for inhibiting hKv4.3 were 7.9, 6.1, 3.9, and 3.2 µM, respectively, at 0.2, 0.5, 1, and 3.3 Hz. The mutagenesis study revealed that the hKv4.3 mutants T366A and T367A in the P-loop helix, and V392A, I395A and V399A in the S6-segment had a reduced channel blocking efficacy of acacetin (IC_50_, 44.5 µM for T366A, 25.8 µM for T367A, 17.6 µM for V392A, 16.2 µM for I395A, and 19.1 µM for V399A). These results demonstrate the novel information that acacetin may inhibit the closed channels and block the open state of the channels by binding to their P-loop filter helix and S6 domain. The use- and rate-dependent blocking of hKv4.3 by acacetin is likely beneficial for managing atrial fibrillation.

## Introduction

It is well recognized that the 4-aminopyridine- (4-AP-) sensitive transient outward potassium current I_to_ is expressed in cardiomyocytes from mouse [1], [2], rat [3], rabbit [4], ferret [5], cat [6], canine [7], and human [8], but not in cardiomyocytes from guinea pig [9] and pig hearts [10], [11]. I_to_ is heterogeneously expressed in transmural ventricular wall of the hearts in human and dogs, determines the morphologies of cardiac action potentials, and generates the prominent phase 1 repolarization and “spike and dome” profile of ventricular epicardial and midmyocardial myocytes in these species [7], [12]. In human and canine hearts, I_to_ is principally encoded by Kv4.3 (*KCND3*) gene [13], [14]. Recent studies have demonstrated that Brugada syndrome-associated I_to_ gain-of-function mutations in *KCND3*-encoded Kv4.3 is believed to mediate an alteration of transmural voltage gradient (epicardium > endocardium), and result in a net outward shift in current and heterogeneous loss of the action potential dome, ST segment elevation on electrocardiogram (ECG), and the development of potentially fatal polymorphic ventricular tachycardia or ventricular fibrillation via phase II reentry [15].

Our previous study [16] has demonstrated the natural flavone acacetin, in addition to blocking human atrial ultra-rapidly-delayed rectifier potassium current (I_Kur_) and acetylcholine-activated potassium current (I_K.ACh_), effectively inhibits human atrial I_to_. This compound increased the atrial effective refractory period and prevented the occurrence of atrial fibrillation in anesthetized dogs without prolonging the QT interval [16]. Our recent study has shown that the natural flavone acacetin is an open channel blocker of hKv1.5 channels with use- and frequency-dependent blocking properties by binding to the S6 domain of the channels [17]. The present study was designed to investigate the properties and molecular determinants of acacetin for inhibiting hKv4.3 channels with whole-cell patch voltage-clamp and mutagenesis approaches.

## Materials and Methods

### Cell line culture and gene transfection

The HEK 293 cell line [18] stably expressing the human Kv4.3 (*KCND3*) gene kindly provided by Dr. Klaus Steinmeyer (Sanofi-Aventis Deutschland GmbH) was maintained in Dulbecco's modified eagle's medium (DMEM, Invitrogen, Hong Kong) supplemented with 10% fetal bovine serum and 400 µg/mL G418 (Sigma–Aldrich). Cells used for electrophysiology recording were seeded on a glass cover slip.

Polymerase chain reaction-based site-directed mutagenesis was used to produce mutations of the pCDNA3.1/hKv4.3 plasmid. Primers used to generate the channel mutants were synthesized by the Genome Research Center, the University of Hong Kong (Hong Kong), and the mutants were generated using a QuikChange kit (Stratagene, La Jolla, CA), and confirmed by DNA sequencing. The mutant was transiently expressed with 4 µg of hKv4.3 mutant cDNA plasmid using 10 µl of Lipofectamine 2000 to determine the mutant hKv4.3 currents.

### Drugs and solutions

Acacetin synthesized in the laboratory as described previously in the US patent (http://www.patentstorm.us/patents/7816400.html) [19] was used in the present study. The compound was dissolved in dimethyl sulfoxide (DMSO) as a 100 mM stock solution. Aliquot stock was stored at –20°C. Tyrode's solution contained (in mM) 140 NaCl, 5.4 KCl, 1 MgCl_2_, 1 CaCl_2_, 10 HEPES, 10 glucose; pH was adjusted to 7.3 with NaOH. The pipette solution contained (in mM) 20 KCl, 110 K-aspartate, 1 MgCl_2_, 10 HEPES, 5 EGTA, 0.1 GTP, 5 Na-phosphocreatine, and 5 Mg-ATP; pH was adjusted to 7.2 with KOH.

### Patch-clamp recording

The coverslips with adherent HEK-hKv4.3 cells on the surface were transferred to an open cell chamber mounted on the stage of an inverted microscope and superfused at 2–3 mL/min. The whole-cell patch-clamp technique was used for electrophysiological recording as described previously [17], [18]. Borosilicate glass electrodes (1.2-mm OD) were pulled using a Brown-Flaming puller (model P-97, Sutter Instrument Co, Novato, CA, USA). They had tip resistances of 2–3 MΩ when filled with the pipette solution. Membrane currents were recorded in voltage-clamp mode using an EPC 10 amplifier and Pulse software (HEKA, Lambrecht, Germany). A 3-M KCl-agar salt bridge was used as the reference electrode. The tip potential was zeroed before the patch pipette touched the cell. After a gigaohm seal was obtained, the cell membrane was ruptured by gentle suction to establish the whole-cell configuration. The series resistance (Rs) was 3–5 MΩ and was compensated by 50–70% to minimize voltage errors, and membrane capacitance was electrically compensated. Liquid junction potential (15.7 mV, calculated with Clampex 9.2) between pipette solution and bath solution was not adjusted during the experiments. Current signal was sampled at 10 kHz, recorded and stored in the hard disk of an IBM compatible computer. The experiments were conducted at room temperature (22–23°C).

### Data analysis

The results are expressed as mean±SEM. Non-linear curve fitting was performed with Pulsefit (HEKA) and Sigmaplot 10.0 (SPSS, Chicago, Ill). Statistical comparisons were analyzed by Student's *t* test for two group data or one-way ANOVA followed by Tukey's test was used for multiple groups. P values less than 0.05 were considered to indicate statistically significant differences.

## Results

### Inhibition of hKv4.3 current by acacetin

**Figure 1:**
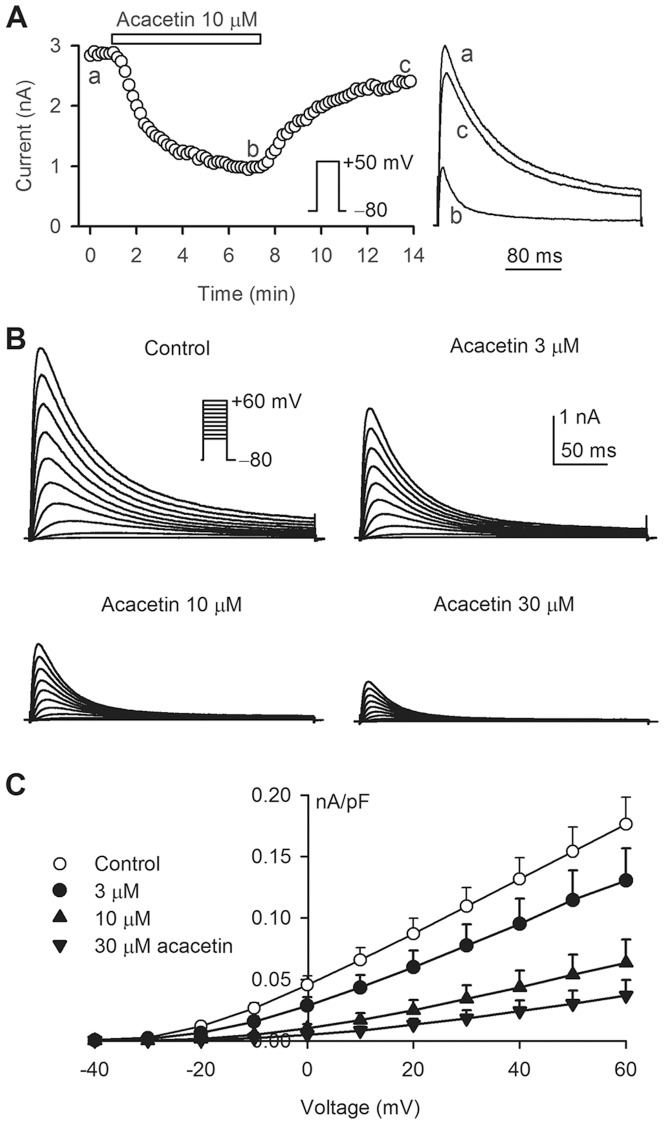
Inhibition of hKv4.3 current by acacetin. **A**. Time course of hKv4.3 step current recorded in a representative HEK 293 cell stably expressing *KCND3* gene in the absence and presence of 10 µM acacetin with a 300-ms test pulse from –80 to +50 mV (inset). Original current traces at corresponding time points are shown in the right of the panel. **B.** Voltage-dependent hKv4.3 current traces recorded in another cell using the protocol as shown in the inset in the absence (control) and presence of 3, 10, and 30 µM acacetin (8 min for each concentration). **C.** Current-voltage (*I*–*V*) relationships of hKv4.3 current in the absence and presence of 3, 10 and 30 µM acacetin (n = 12, P<0.05 or P<0.01 vs. control at −10 to +60 mV).

Figure 1A illustrates the time course of hKv4.3 current recorded in a representative cell, in the absence and presence of 10 µM acacetin, using a 300-ms voltage step to +50 mV from a holding potential of −80 mV (inset, 0.2 Hz). Acacetin gradually inhibited the hKv4.3 current. The current amplitude was measured from zero to the current peak. The inhibitory effect significantly recovered on washout. Similar results were obtained in eight other cells.

Figure 1B displays the voltage-dependent hKv4.3 current determined in a typical experiment with the voltage protocol shown in the inset, in the absence and presence of acacetin. The current was inhibited by 3, 10, or 30 µM acacetin in a concentration-dependent manner. Figure 1C shows the current-voltage (*I*–*V*) relationships of hKv4.3 current during control and after application of 3, 10, and 30 µM acacetin. The current was significantly inhibited by acacetin (n = 15, P<0.05 or P<0.01 vs. control at 0 to +60 mV).

**Figure 2:**
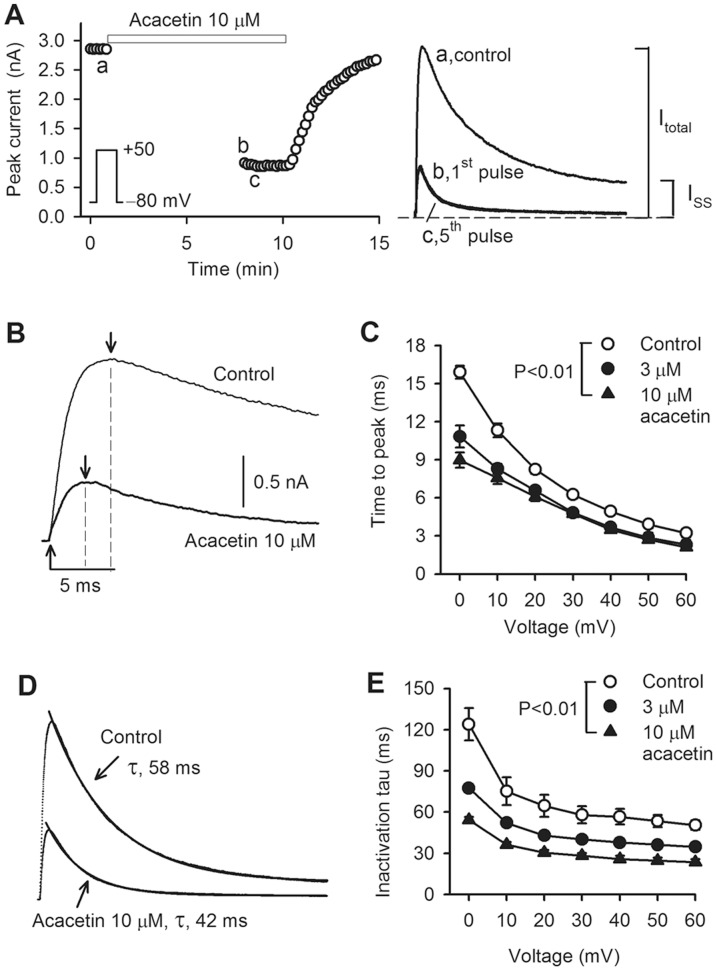
Blocking properties of hKv4.3 channels by acacetin. **A.** Time course of hKv4.3 current recorded in a representative cell with the voltage step shown in the inset during control and after 10 µM acacetin superfusion for 6 min without the voltage pulse depolarization and for 2 min with the voltage pulse depolarization, then drug washout. The currents recorded at corresponding time points are shown in the right of the panel. The arrows indicate the time to peak of the current activation. I_total_, total current; I_SS_, steady-state (or sustained) current. **B.** Expanded current traces of hKv4.3, showing the measurement of the time to peak of hKv4.3 current. **C.** Mean values of the time to peak of the current activation at 0 to +60 mV before and after application of 3 and 10 µM acacetin (n = 10 experiments, P<0.01 vs. control). **D.** Inactivation of hKv4.3 current was fitted to a monoexponetial equation with the time constants shown before (control) and after 10 µM acacetin. **E.** Mean values of time constant of hKv4.3 current inactivation at 0 to +60 mV before and after application of 3 and 10 µM acacetin (n = 10 experiments, P<0.01 vs. control).

To analyze the blocking properties of hKv4.3 channels, a 300-ms voltage step to +50 mV from a holding potential of −80 mV was used to record the current before acacetin application (10-s interval), and then discontinued during six min of 10 µM acacetin administration (Fig. 2A) at a holding potential −80 mV to ensure that all channels were in the closed state. The blocking effect of hKv4.3 channels by acacetin was evaluated by reapplying the protocol after the six min of exposure. Remarkable suppression of hKv4.3 current by acacetin was observed at 1^st^ pulse of the reapplied voltage step (Fig. 1A). No significant difference was observed between the current recorded at 1^st^ pulse and those recorded by the following pulses. The channel blockade was reversed by drug washout. Similar results were obtained in other four cells.

It should be noted that inhibitory effect of acacetin on hKv1.5 current increases at following pulses after the 1^st^ pulse of reapplied voltage step by binding to the open channels [17]. No difference for the inhibiting effect on the 1^st^ pulse-hKv4.3 current and the currents activated by following pulses suggests that acacetin might inhibit the closed channels. However, it is generally believed that the closed channel blocker 4-aminopyridine slowed the inactivation process and decreased the time to peak current in Kv4.2 channel current expressed in *Xenopus* oocytes and transient outward potassium current (I_to_) in ferret cardiac myocytes, and induced a ‘crossover phenomena’ of the current [5], [20]. However, acacetin clearly facilitated hKv4.3 current inactivation (Fig. 1A and 1B), reduced the time to peak current, and also induced a strong inhibition of steady-state (or sustained) current (I_SS_) (right panel of Fig 2A). This suggests that acacetin likely inhibit the current by binding to both the closed and open channels.

To analyze the open channel blocking property, hKv4.3 traces were expanded to measure the time to peak of hKv4.3 channel activation before and after application of 10 µM acacetin (Fig. 2B). The mean values of the voltage-dependent time to peak of the channel were significantly reduced by 3 or 10 µM acacetin at all test potentials (Fig. 2C). Figure 2D shows that hKv4.3 current was well-fitted to a monoexponential function with the time constants shown before and after 10 µM acacetin. The inactivation time constant of Kv4.3 current was significantly reduced by 3 or 10 µM acacetin at all test potentials (0 to +60 mV, n = 10, P<0.01 vs. control). These results support the notion that acacetin also inhibits hKv4.3 current by blocking the open channel.

### Effects of acacetin on kinetics of hKv4.3 current

**Figure 3:**
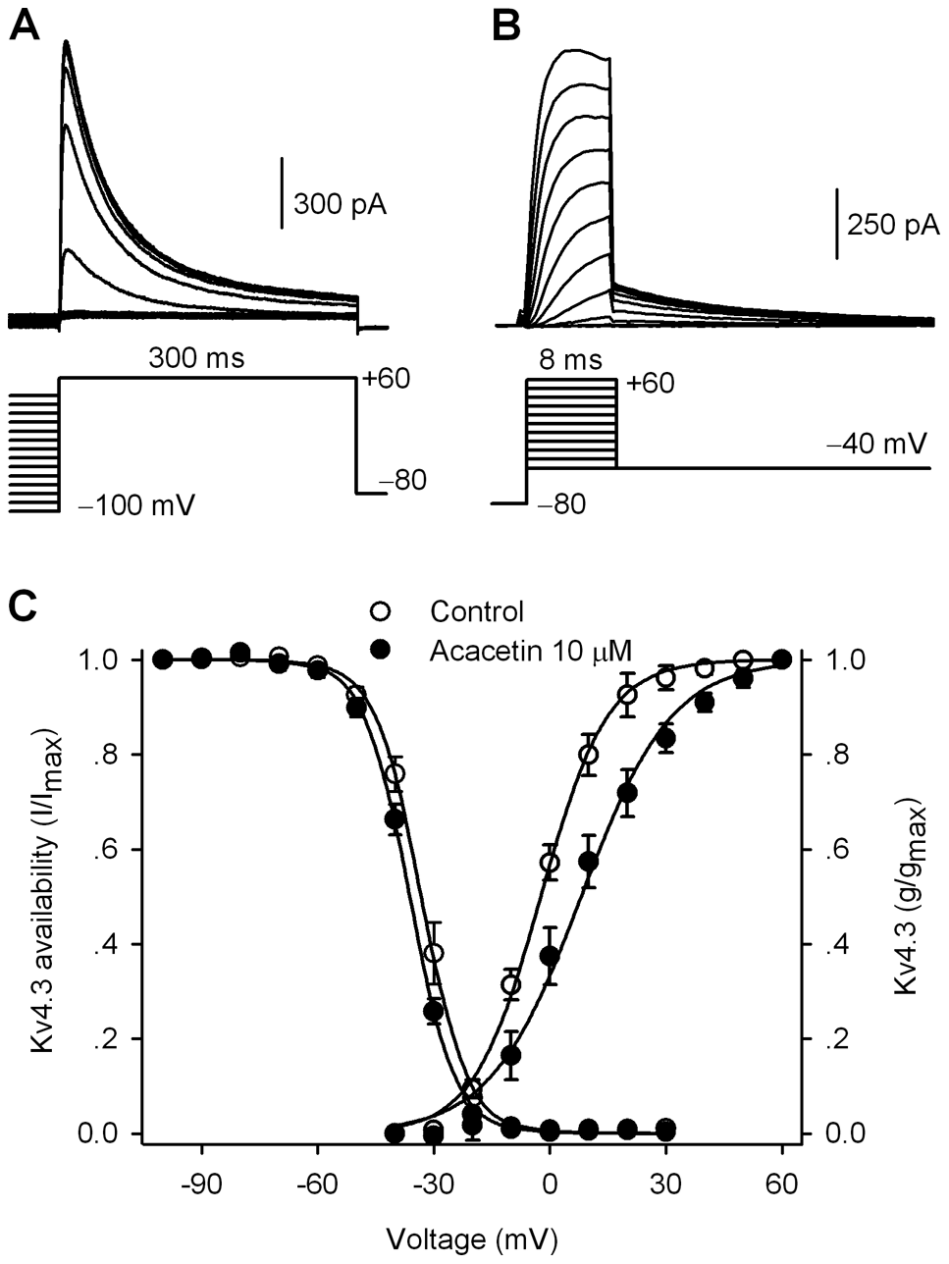
Effects of acacetin on voltage-dependent kinetics of hKv4.3 current. **A.** Protocol and current traces used to assess availability (I/I_max_, steady-state inactivation) of hKv4.3 current. **B.** Protocol and tail current traces used to assess activation conductance (g/g_max_, steady-state activation) of hKv4.3 current. **C.** Mean values of hKv4.3 current (I/I_max_) variables and conductance (g/g_max_) variables before and after 10 µM acacetin were fitted to the Boltzmann function: g = 1/(1+exp((V_1/2_−V_t_)/K)), where V_1/2_ is the voltage of 50% channel availability or maximal activation of the channel, V_t_ is the test potential, and K is slope factor.

Figure 3A shows the representative current and voltage protocol used for determining the availability (I/I_max_) of hKv4.3 current. Figure 3B illustrates the tail current recorded by the voltage protocol for determining the steady-state activation (g/g_max_) of the channel. The variables (Fig. 3C) of I/I_max_ and g/g_max_ were fitted to a Boltzmann function in individual cells as described previously [12]. The V_1/2_ of hKv4.3 current availability was not significantly changed (−31.3±1.7 mV in control, and −35.7±1.1 mV in 10 µM acacetin, n = 8, P = NS vs. control), while the V_1/2_ of activation conductance was positively shifted by 10.1 mV (−1.7±1.8 mV in control, 8.4±2.9 mV in acacetin, n = 9, P<0.01 vs. control). This effect was not observed in human atrial I_to_ [16], and this difference may be related to the lack of the regulatory β-subunits KChIPs in HEK 293 cells.

**Figure 4:**
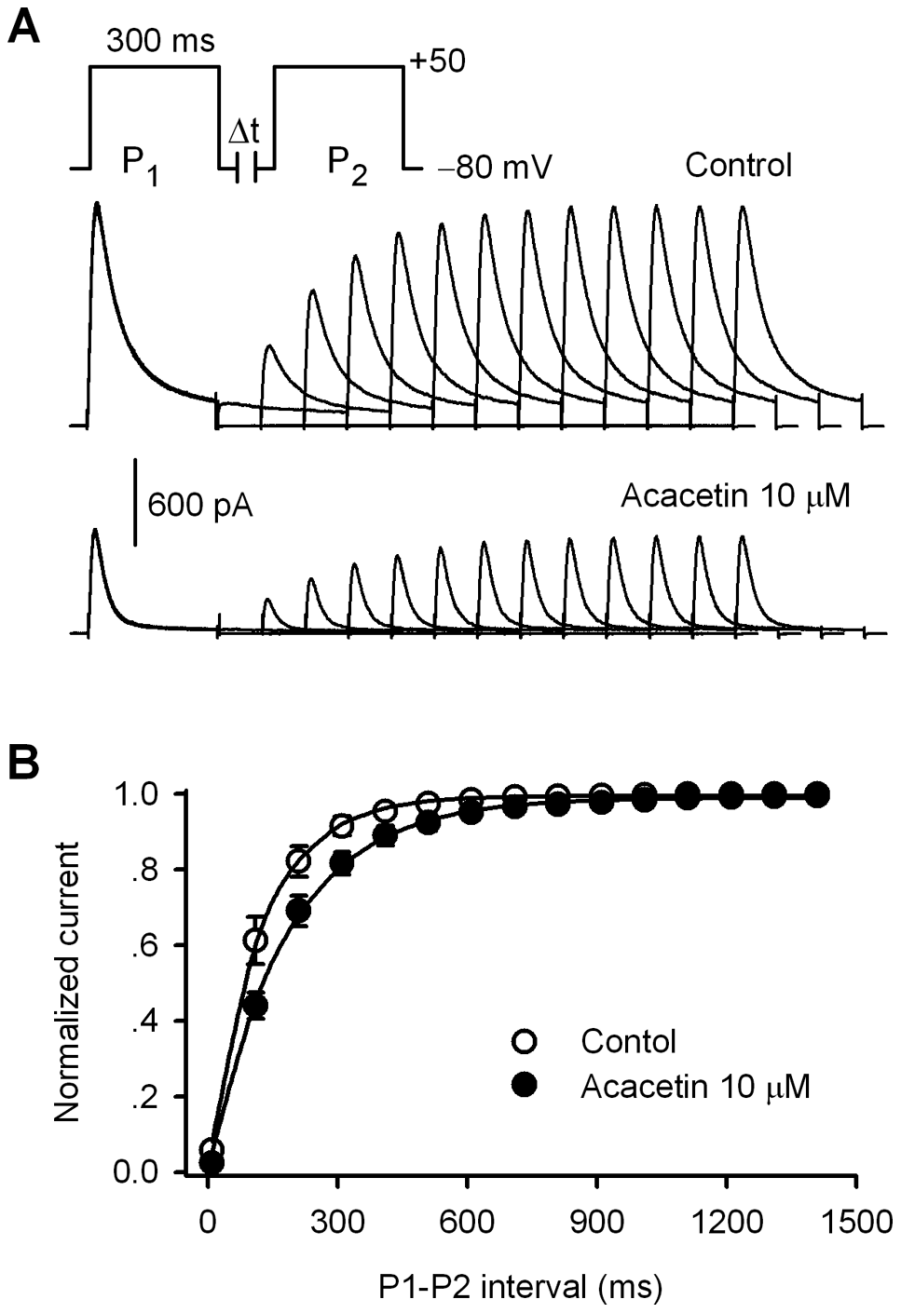
Effect of acacetin on recovery of hKv4.3 current from inactivation. **A.** Protocol and hKv4.3 current traces recorded in a representative cell before (control) and after 10 µM acacetin (8 min) used to assess the time constant of recovery of the channel from inactivation. **B.** Mean values of recovery time course of hKv4.3 current from inactivation were fitted to a mono-exponential function before and after application of 10 µM acacetin.

The effect of acacetin on the recovery kinetics of hKv4.3 current was determined with a paired pulse using a 300-ms step to +50 mV from a holding potential of −80 mV with variable P1–P2 interval as shown in the inset of Fig. 4A. Acacetin (10 µM) reduced the current amplitude and slowed the recovery of hKv4.3 current from inactivation. The recovery time course was fitted to a monoexponential function in individual cells before and after application of 10 µM (Fig. 4B). The time constant (τ) of recovery from inactivation of hKv4.3 current was increased from 112.7±13.6 ms in control to 188.2±19.5 ms after 10 µM acacetin (n = 9, P<0.01 vs. control). The slowed recovery of hKv4.3 current from inactivation was similar to the observation in human atrial I_to_ [16].

### Use- and frequency-dependent block of hKv4.3 channels by acacetin

**Figure 5:**
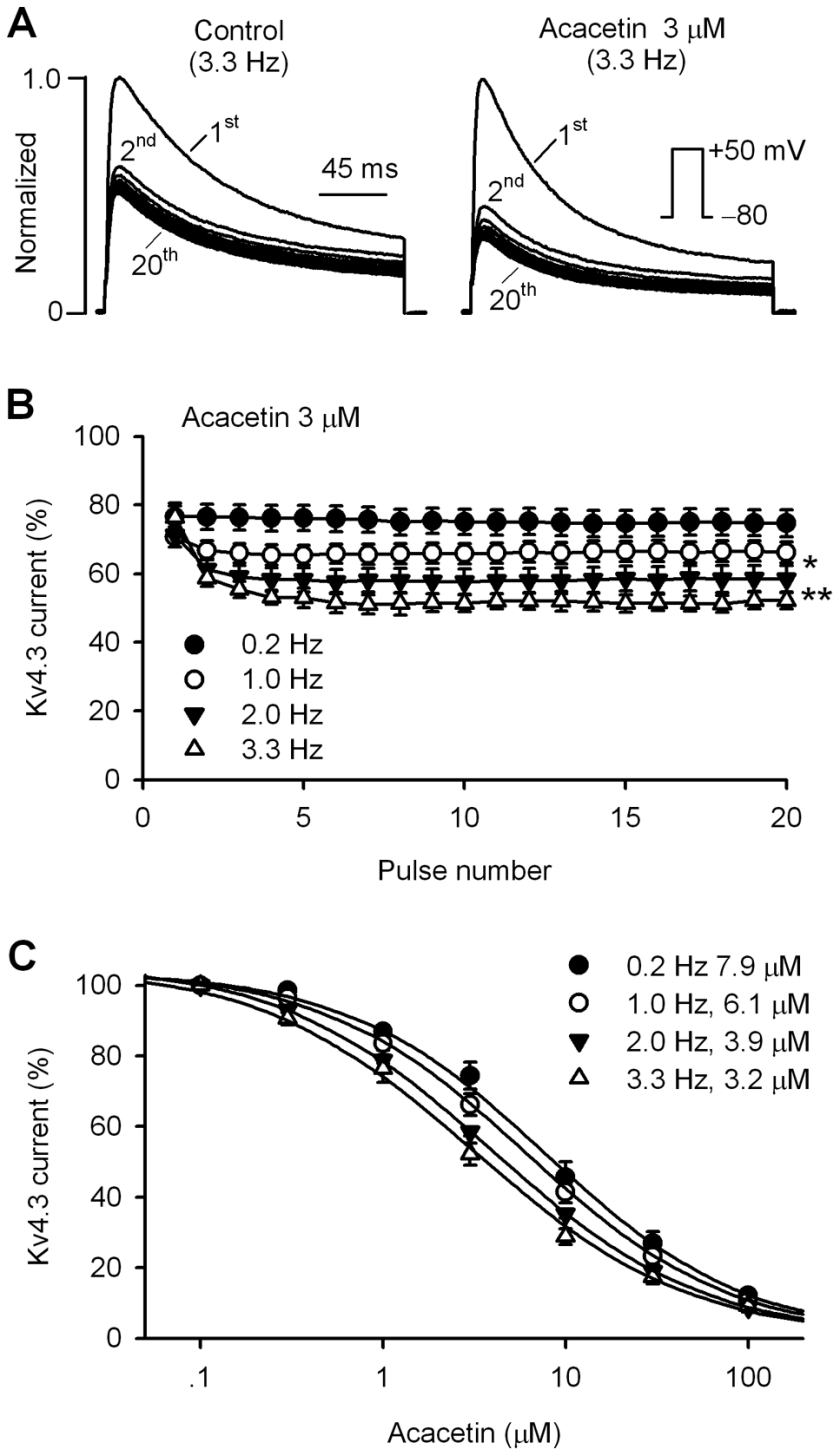
Use- and frequency-dependent inhibition of hKv4.3 current by acacetin. **A.** hKv4.3 current traces recorded in a representative cell with a 200-ms pulse at 3.3 Hz before (control) and after application 3 µM acacetin. **B**. Mean percentage values of use-dependent inhibition of hKv4.3 current (at +50 mV) by 3 µM acacetin at 0.2, 1, 2, and 3.3 Hz. **C.** Concentration-response relationship curves of acacetin for inhibiting hKv4.3 current at 20^th^ pulse were fitted to Hill equation to obtain IC_50_ (n = 7–15 experiments for each concentration or frequency) at frequencies of 0.2–3.3 Hz.

The slowed recovery of hKv4.3 channels from inactivation suggests that blockade of hKv4.3 channels may be use- and frequency-dependent. The use-dependent blockade of hKv4.3 channels by acacetin was determined at 0.2, 1, 2, and 3.3 Hz using a train of 20 pulses of a 200-ms voltage step. Figure 5A shows the normalized hKv4.3 current traces recorded at 3.3 Hz in a representative cell before and after application of 3 μM acacetin. Though hKv4.3 current showed a significant use-dependent inhibition in control, the use-dependent blockade was evident with acacetin. The current blockade at the first pulse was less than that at following pulses. The fractional blockade of hKv4.3 current at each frequency with 3 μM acacetin is illustrated in Fig. 5B. The use-dependent blockade was enhanced as the depolarization frequency was increased. In addition, acacetin exhibited more potent of inhibition on hKv4.3 current at 3.3 Hz than that at 0.2 Hz and 1.0 Hz. Figure 5C shows the percentage current at the 20^th^ pulse of different frequencies with 0.1–100 µM acacetin. The curves were fitted to a Hill equation to obtain the IC_50_. The IC_50_ of acacetin for inhibiting hKv4.3 current was reduced as increase of the depolarization frequency (IC_50_: 7.9, 6.1, 3.9, and 3.2 μM at 0.2, 1.0, 2.0, and 3.3 Hz, respectively).

### Effect of acacetin on closed-state inactivation of hKv4.3 current

The steady-state inactivation of Kv4.3 channels occurs predominantly from the closed state [21], [22], here we determined whether acacetin would affect the development kinetics of closed-state inactivation of hKv4.3 channels. Acacetin (10 µM) slightly accelerated the closed-state inactivation of hKv4.3 channels (Figure S1). The closed-state inactivation time constant was 1683.3±134.1 ms in control, and 1355.2±59.2 ms in 10 µM acacetin (n = 6, P<0.05 vs. control). The result suggests that acacetin may accelerate the kinetics of closed-state inactivation of hKv4.3 channels.

### Molecular determinants of hKv4.3 channel blockade by acacetin

**Figure 6:**
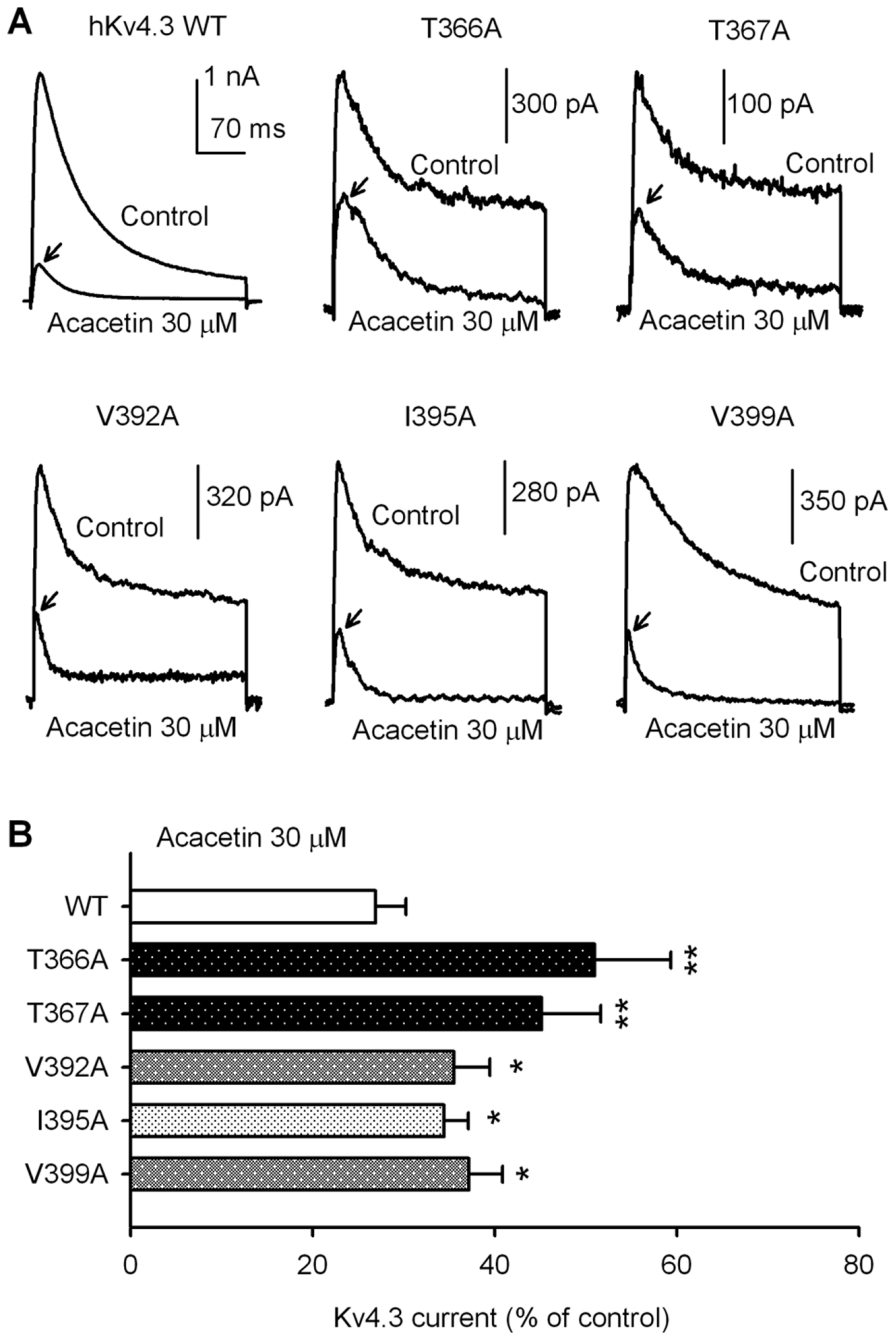
Effects of acacetin on WT and mutant hKv4.3 currents. **A.** Current traces recorded in HEK 293 cells expressing WT, T366A, T367A, V392A, I395A, and V399A hKv4.3 channels, respectively, with a 300-ms voltage step to +50 mV from a holding potential of −80 mV before (control) and after 30 µM acacetin treatment for 5 min. The arrows indicate the current inhibition levels. **B.** Mean percent inhibition of WT and mutant hKv4.3 currents by 30 µM acacetin (n = 12 for control, n = 5–7 for each mutant; *P<0.05, **P<0.01 vs. WT).

**Figure 7:**
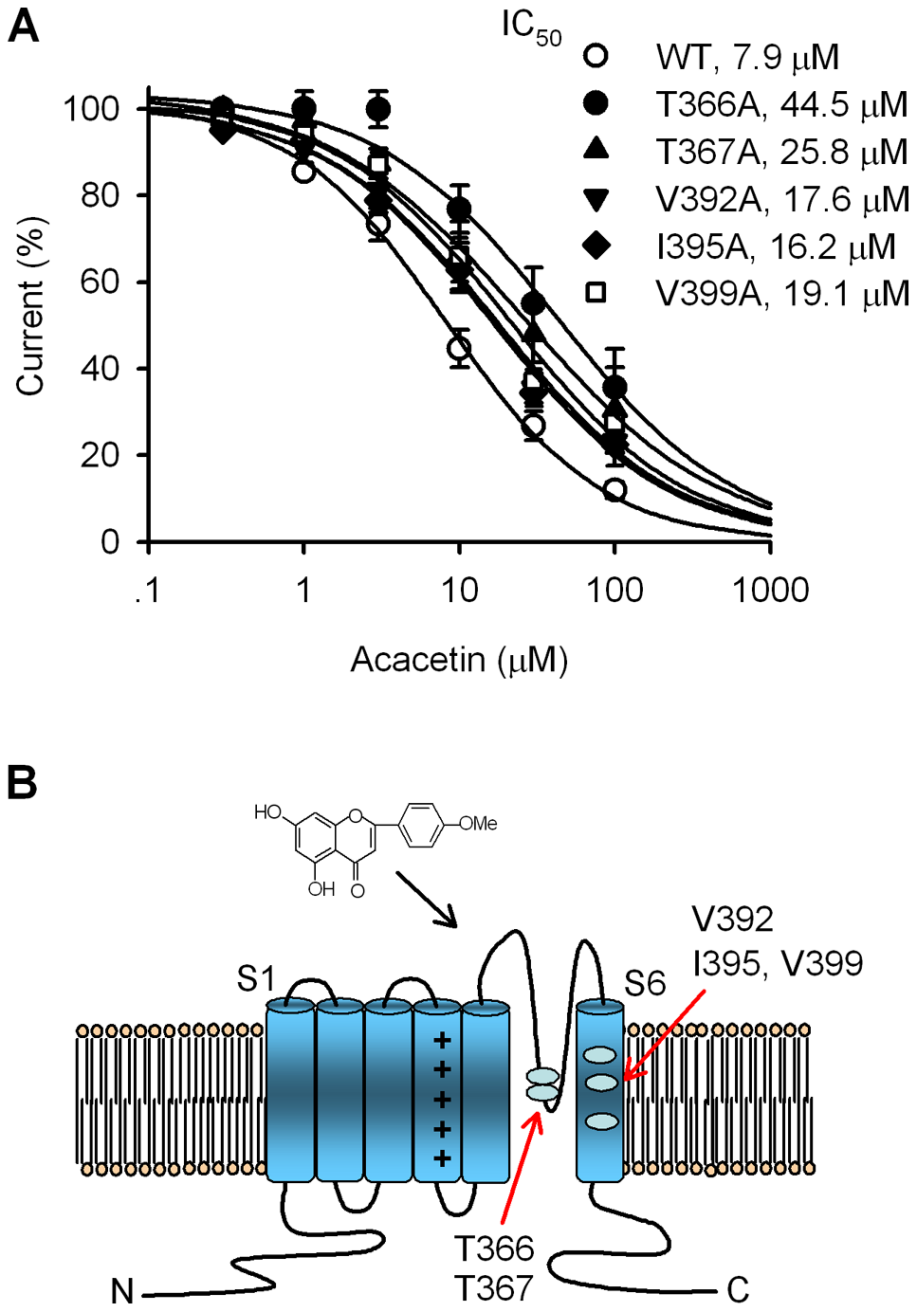
Molecular determinants of hKv4.3 channel block by acacetin. **A.** Concentration-response relationship curves were fitted to the Hill equation to obtain the IC_50_s of acacetin for inhibiting WT and mutant hKv4.3 channels as shown in the inset (n = 5–12 for each concentration). **B.** Schematic graph showing the putative binding sites of acacetin at T366, T367 in the P-loop helix and V392, I395, and V399 in the S6-segment of Kv4.3 channels.

The molecular determinants of the blockade of hKv4.3 channels by acacetin were investigated using the mutants T366A and T367A in the P-loop helix, and V392A, I395A, and V399A in the S6 transmembrane domain. Figure 6A shows the representative current traces of wild type (WT), T366A, T367A, V392A, I395A, and V399A hKv4.3 channels activated with a 300-ms voltage step to +50 mV from a holding potential of −80 mV in the absence and presence of acacetin (30 µM). Acacetin at 30 µM markedly blocked the WT hKv4.3 current. Less inhibition was observed for the T366A, T367A, V392A, I395A, and V399A currents. The mean values of percentage inhibition of hKv4.3 currents are illustrated in Fig. 7B. Acacetin at 30 µM inhibited the WT hKv4.3 current by 74.2±3.3% (n = 12), T366A by 49.1±8.3% (n = 8, P<0.01 vs. WT), T367A by 54.9±6.5% (n = 7, P<0.01 vs. WT), V392A by 64.5±3.9% (n = 9, P<0.05 vs. WT), I395A by 65.6±2.7% (n = 8, P<0.05 vs. WT), and V399A by 62.9±3.7% (n = 6, P<0.05. vs. WT), respectively.

The concentration-dependent response to acacetin was evaluated in WT and mutant hKv4.3 channels, and the resulting curves were fitted to a Hill equation as in Fig. 7A. The IC_50_s (at 0.2 Hz) of acacetin in inhibiting hKv4.3 currents were 7.9 µM for WT, 44.5 µM for T366, 25.8 µM for T367A, 17.6 µM for V392A, 16.2 µM for I395A, and 19.1 µM for V399A, respectively. These results suggest that T366 and T367 in the P-loop helix, V392, I395, and V399 in the S-6 segment are the molecular determinants of channel blocking by acacetin (Fig. 7B).

## Discussion

The present study demonstrates that the natural flavone acacetin inhibits hKv4.3 channels stably expressed in HEK 293 cells in a use- and frequency-dependent manner by binding to not only the open state of the channels, but also the closed channels. The effect of acacetin for blocking hKv4.3 current was enhanced as the stimulus frequency was increased from 0.2 Hz (IC_50_ = 7.9 µM) to 3.3 Hz (IC_50_ = 3.2 µM). The efficacy at 0.2 Hz is close to that for inhibiting human atrial I_to_ (IC_50_ = 9.3 µM) [16].

**Figure 8:**
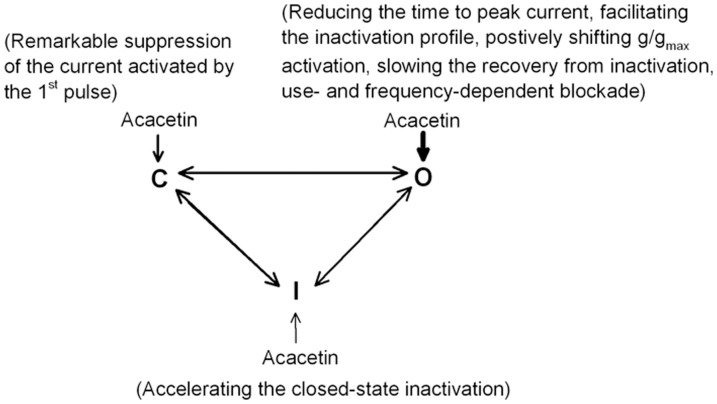
Blocking scheme graph shows that acacetin inhibits hKv4.3 current by interaction with different states of the channel. C, closed states; O, open states; I, inactivated states. The thickness of the arrows suggests the estimated potency of acacetin for different states of the channel.

In addition to the use- and frequency-dependent effect, the open channel blocking property of acacetin was reflected in the reduced time to peak of the current activation and the decreased time constant of Kv4.3 current inactivation. This indicates that acacetin may quickly bind to the channels when they open. The open channel property of acacetin is further supported by the slowed recovery of hKv4.3 channels from inactivation and the positive shift of g/g_max_ of the channel activation. This is different from the Kv4.3 blocker allitridi that also binds to the open state of the channel, but does not show a slowed recovery from inactivation and use- and frequency-dependent effect [23], which may be related to that acacetin is not a pure open channel blocker for hKv4.3 channels. Acacetin also inhibits the closed channels, which is reflected in the remarkable suppression of the current activated by the 1^st^ pulse of reapplied voltage steps after administration of acacetin. This property is different from that in blocking open channels of hKv1.5 [17]. The blockade of hKv4.3 and hKv1.5 channels by acacetin is likely from cytoplasmic surface, because both hKv4.3 current and hKv1.5 current were not significantly inhibited by intracellular dialysis with the patch pipette solution containing 10 µM acacetin (the authors' unpublished observations). Therefore, the intrinsic inactivation gating (i.e. ball and chain) of hKv4.3 channels may not be affected by acacetin. In addition, acacetin slightly accelerated the closed-state inactivation of the channel. These are illustrated in the blocking scheme (Fig. 8).

Mutagenesis experiments revealed that the inhibitory efficacy of acacetin on the hKv4.3 mutants T366A and T367A of the P-loop of the pore helix was significantly reduced. This implies that acacetin may be trapped into the channel pore and block the open channel. Moreover, the mutants V392A, I395A, and also V399A, of the S6 domain exhibit a significantly reduced response to acacetin, indicating that in addition to binding to the P-helix filter, acacetin may interact with V392, I395, and V399 of the S6 domain. Therefore, the five residues T366, T367, V392, I395, and V399 of the channel are involved in the inhibition of hKv4.3 current by acacetin. These sites are the equivalent residues of T479, T480, V505, I508, and V512 of hKv1.5 channels, respectively [17]. However, the blocking binding sites of acacetin for blocking Kv4.3 channels are slightly different from those for blocking Kv1.5 channels where the P-loop helix (e.g. T480) is not involved in the binding of acacetin [17].

It is generally believed that I_to_ is relatively larger in the atrial cells than that in the ventricular cells, so that inhibition of I_to_ may cause a prolongation of repolarization predominantly in the atria more than that in the ventricle [24]. Human cardiac I_to_ (or Kv4.3) is considered to be a target for developing anti-atrial fibrillation drugs [24], [25]. Acacetin inhibited hKv4.3 current, especially at high frequencies. Although the blockade of hKv4.3 channels by acacetin is relatively weaker than that of hKv1.5 channels, the combination with its frequency-dependent blockade of hKv1.5/I_Kur_ [17], favors the prolongation of atrial action potential duration and/or effective refractory period in human atrial myocytes, which benefits for anti-atrial fibrillation. This effect has been observed in experimental canine model [16].

An increase of I_to_ has been found to be involved in genesis of cardiac ventricular arrhythmias or Brugada syndrome [15], [26]–[28]. Because I_to_ plays a crucial role in phase 1 fast repolarization of ventricular action potentials, especially in the midmyocardium and epicardium in humans [8], [12], [29] and in dogs [7]. Up-regulation of I_to_ is involved in generation of Brugada syndrome and idiopathic ventricular fibrillation [30] by shifting cardiac repolarization and inducing J-wave syndromes that triggers the life-threatening arrhythmia [15], [31]. It has been documented that an increase of I_to_ amplitude by gain-of-function mutations in the *KCND3*-encoded Kv4.3 channels is the molecular pathogenesis for the lethal arrhythmia in patients with Brugada syndrome [30]. Suppression of ventricular arrhythmia in Brugada patients with quinidine [32], [33] is believed to be related to its I_to_ blocking effect [34]–[36].

A recent study has reported that oxidation stress increases I_to_ conductance and promotes EADs (early after-depolarizations) by setting myocardial action potential plateau into the voltage range where I_Ca,L_ reactivation is facilitated and I_Ks_ activation is slowed, and the EADs are suppressed by the I_to_ blocker 4-AP [28]. It should be noted that I_to_ inhibition by 4-AP may restore the epicardial action potential dome, reduce both transmural and epicardial dispersion of repolarization, normalize the ST segment, and prevent phase 2 reentry and ventricular tachycardia/ventricular fibrillation in experimental Brugada syndrome [15], [31], [37]. These studies suggest that I_to_ blockade also has a therapeutic effect on ventricular arrhythmias resulted from oxidation-induced EADs or Brugada syndrome. Acacetin has a strong inhibition of hKv4.3 current, and may also be a candidate drug in suppressing ventricular arrhythmias related to Brugada syndrome. Although acacetin also blocks hKv1.5 channels, this effect would not affect other phases of action potential in human ventricles, because no functional hKv1.5/I_Kur_ is present in ventricular myocytes of human hearts [38]. Blockade of hKv4.3 by acacetin would favor the correction of Brugada's abnormal repolarization at early phase (phase 1) action potential in human ventricles; however, this remains further investigation in the future.

Collectively, the present study demonstrates that acacetin mainly blocks hKv4.3 channels by binding to open states and interacting with T366 and T367 in the P-loop helix and V392, I395, and V399 within the S6 domain. The use- and frequency-dependent blocking property of the hKv4.3 channels and also hKv1.5 channels suggest that this natural flavone compound could strongly inhibit atrial fibrillation. Further effort is required to study whether it is effective in managing ventricular arrhythmias related to Brugada Syndrome.

## Supporting Information

Figure S1 **Effect of acacetin on closed-state inactivation of hKv4.3 channels.**
(PDF)
