# Correction: Properties and Molecular Determinants of the Natural Flavone Acacetin for Blocking hKv4.3 Channels

**DOI:** 10.1371/annotation/caf130c3-5026-41cd-9dda-5eac7c0f016f

**Published:** 2013-10-21

**Authors:** Hui-Jun Wu, Hai-Ying Sun, Wei Wu, Yan-Hui Zhang, Guo-Wei Qin, Gui-Rong Li

There are multiple errors present in this work. 

The email for Corresponding Author Gui-Rong Li is incorrect. It should be grli@hkucc.hku.hk.

There are two errors in the "Inhibition of hKv4.3 current by acacetin" portion of the Results section. 

-There is an error in the last line of the third paragraph. The correct sentence reads: "The current was significantly inhibited by acacetin (n= 15, P<0.05 or P<0.01 vs. control at -10 to +60 mV)." 

-There is an error in the penultimate sentence of the last paragraph. Figure 2E should be referenced following the word "constant." The sentence should read: "The inactivation time constant (Fig. 2E) of Kv4.3 current was significantly reduced by 3 or 10 μM acacetin at all test potentials (0 to +60 mV, n= 10, P<0.01 vs. control).

There are two errors in the “Molecular determinants of hKv4.3 channel blockade by acacetin” portion of the Results section. 

- The penultimate sentence of the first paragraph should reference Figure 6B and not Figure 7B. The correct sentence reads: “The mean values of percentage inhibition of hKv4.3 currents are illustrated in Fig. 6B.”

- In the second sentence of the second paragraph, T366A is incorrectly marked T366. The correct sentence reads: The IC_50_s (at 0.2 Hz) of acacetin in inhibiting hKv4.3 currents were 7.9 μM for WT, 44.5 μM for T366A, 25.8 μM for T367A, 17.6 μM for V392A, 16.2 μM for I395A, and 19.1 μM for V399A, respectively.

There is an error in the legend for Figure 2A. The correct legend reads: "Time course of hKv4.3 current recorded in a representative cell with the voltage step shown in the inset during control and after 10 μM acacetin superfusion for 6 min without the voltage pulse depolarization and for 2 min with the voltage pulse depolarization, then drug washout. The currents recorded at corresponding time points are shown in the right of the panel. I_total_, total current; I_SS_, steady-state (or sustained) current.”

There is an error in the legend for Figure 6B. The correct legend reads: “Mean percent inhibition of WT and mutant hKv4.3 currents by 30 μM acacetin (n = 12 for WT, n = 5–7 for each mutant; *P<0.05, **P<0.01 vs. WT).

