# Supplementary material for: Properties and Molecular Determinants of the Natural Flavone Acacetin for Blocking hKv4.3 Channels
Source: PLoS One. 2013 Mar 20;8(3):e57864. doi: 10.1371/journal.pone.0057864 (PMC3603988; doi:10.1371/journal.pone.0057864)
Supplement: Figure S1 — Effect of acacetin on closed-state inactivation of hKv4.3 channels. (PDF) [file pone.0057864.s001.pdf]

Figure S1

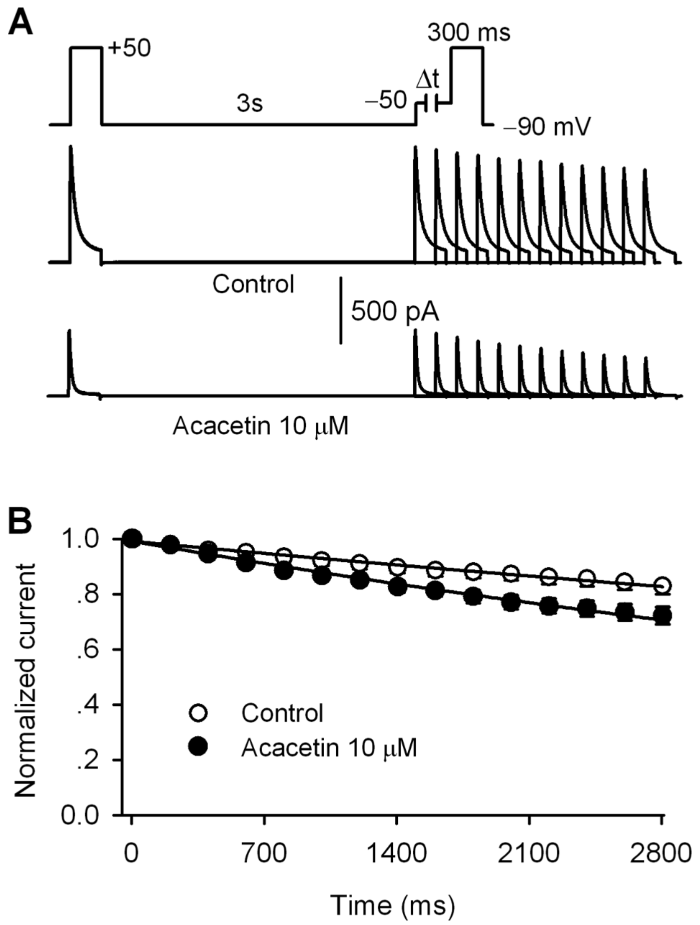

Figure S1. Effect of acacetin on closed–state inactivation of hKv4.3 channels.

A. The hKv4.3 current traces recorded by the voltage protocol (inset) used for determining closed–state inactivation kinetics of the channel in a representative cell before (control) and after 10 μM acacetin (8 min).

B. Mean values (n=6) of time course of the closed–state inactivation of hKv4.3 current was fitted to a monoexpontial equation before (control) and after application of 10 μM acacetin.

The current was recorded with a double pulse (300–ms) protocol. A progressively increasing duration of a closed state potential of −50 mV (below the activation threshold) was applied for second pulse. The current activated by second pulse was normalized and plotted against the time duration of closed–state potential. At 2.8 s duration of closed–state potential, the inactivation of hKv4.3 current was 0.82±0.03 of control before drug administration, and 0.72±0.04 of control after 10 μM acacetin (n=6, P<0.01 vs. control).
